# The Nrf2/HO-1 signaling pathway in arthritis: from molecular mechanisms to therapeutic potential

**DOI:** 10.3389/fcell.2026.1728679

**Published:** 2026-01-21

**Authors:** Lin Zhang, Xianpeng Huang, Huazhang Xiong, Lidan Yang

**Affiliations:** Department of Orthopedics, Affiliated Hospital of Zunyi Medical University, Zunyi, China

**Keywords:** gouty arthritis, Nrf2/HO-1, osteoarthritis, rheumatoid arthritis, therapeutic potential

## Abstract

Arthritis, a group of common diseases characterized by joint inflammation, cartilage destruction, and imbalance in bone remodeling, has high global prevalence and disability rates. In recent years, oxidative stress and chronic inflammation have been widely recognized as core mechanisms jointly driving its pathological process. The antioxidant response axis formed by nuclear factor erythroid 2-related factor 2 (Nrf2) and heme oxygenase-1 (HO-1) plays a key role in maintaining joint tissue redox balance and suppressing excessive inflammatory responses. Extensive basic and translational research indicates that the Nrf2/HO-1 pathway exerts protective effects through multiple mechanisms: reducing reactive oxygen species (ROS) levels, inhibiting nuclear factor-kappa B (NF-κB)-mediated inflammation, regulating macrophage polarization, and influencing processes such as apoptosis, ferroptosis, and fibrosis, thereby significantly alleviating tissue damage and clinical symptoms in arthritis. Currently, various natural products, small-molecule compounds, and drug repurposing strategies targeting the activation or regulation of this pathway have shown promising joint protective effects in animal experiments, suggesting Nrf2/HO-1 is a potential disease-modifying therapeutic target. This review systematically summarizes the latest research progress on the role of Nrf2/HO-1 in the pathogenesis of arthritis, experimental evidence from cellular and animal models, therapeutic strategies targeting this pathway, and discusses key scientific and technical challenges for future clinical translation.

## 1 Introduction

Arthritis is a group of common diseases characterized by chronic joint inflammation, degenerative cartilage changes, and structural bone alterations, significantly impacting patients’ quality of life and posing a substantial societal health burden. Osteoarthritis (OA) and rheumatoid arthritis (RA) are the most prevalent clinical forms. Although their etiologies and pathogenesis differ, oxidative stress and persistent low-grade inflammation are recognized as key pathological drivers jointly promoting joint tissue damage, activation of matrix-degrading enzymes (e.g., matrix metalloproteinases), and cell death. nuclear factor erythroid 2-related factor 2(*NFE2L2*, Nrf2*)* serves as the master transcription factor regulating cellular oxidative stress responses. By binding to the antioxidant response element (*ARE*), it initiates the expression of various detoxification and antioxidant genes, including heme oxygenase-1(*HMOX1*, HO-1) and NAD(P)H quinone oxidoreductase 1(*NQO1*), thereby maintaining intracellular redox homeostasis at the molecular level and curbing excessive activation of inflammatory signals (Sheng et al., 2024a; Lal et al., 2021; Saha, 2024).

Within joint tissues, Nrf2 activation primarily exerts protective effects through two major mechanisms: First, it directly enhances the transcription of various antioxidant enzymes and phase II detoxifying enzymes, effectively reducing ROS and lipid peroxidation products, thereby protecting chondrocytes and synoviocytes from oxidative damage at the source. Second, its key downstream effector, HO-1, catalyzes heme degradation, producing active metabolites such as carbon monoxide (CO) and biliverdin/bilirubin. These molecules possess significant anti-inflammatory, antioxidant, and immunomodulatory functions, promoting macrophage polarization towards the reparative M2 phenotype, thus inhibiting the spread of inflammation and matrix destruction at the tissue microenvironment level (Wang and He, 2022; Song et al., 2021; Ma et al., 2024; Shen P. C. et al., 2023; Zhang J. et al., 2023). Consistent evidence from *in vitro* and *in vivo* experiments confirms that enhancing Nrf2/HO-1 signaling through pharmacological means or genetic manipulation significantly alleviates joint pathology induced by trauma, immune dysregulation, or metabolic disturbances; conversely, inhibiting this pathway often exacerbates inflammatory injury and cartilage degeneration (Hung et al., 2024; Jiang et al., 2022a; Chen et al., 2022; Zhai et al., 2018).

Recent research has further revealed complex cross-talk between Nrf2/HO-1 and other key signaling networks (e.g., NF-κB, NLRP3 inflammasome, PI3K/Akt, MAPK, and pathways related to iron metabolism and ferroptosis). This multi-level interactive network mechanistically explains why single-target therapies often show limited efficacy in arthritis, suggesting that “multi-target” modulation strategies based on Nrf2 may be more beneficial for comprehensively blocking disease progression (Sheng et al., 2024a; Fang et al., 2024; Wei et al., 2022). Furthermore, a growing number of natural small molecules, approved drugs, and novel drug delivery systems have been confirmed to exert joint protective effects by activating Nrf2 or upregulating HO-1 expression, providing a rich pool of candidate resources for developing disease-modifying therapeutic strategies (Sheng et al., 2024a; Wang et al., 2022).

Despite encouraging preclinical evidence, translating Nrf2/HO-1 targeting strategies into safe and effective clinical therapies faces several challenges: First, Nrf2 may act as a “double-edged sword” in different cell types and disease stages, offering protection while potentially participating in metabolic reprogramming and exacerbating damage under certain pathological contexts. Second, the potential safety concerns of long-term systemic Nrf2 activation, particularly regarding metabolic homeostasis and drug-metabolizing enzymes, require systematic evaluation. Additionally, the delivery efficiency and cellular targeting of local joint administration need improvement to reduce off-target effects and enhance treatment precision (Sun et al., 2015; Wang et al., 2023). Accordingly, this article will review the molecular and cellular mechanisms of Nrf2/HO-1 in the arthritic pathological process, summarize key findings from experimental models, evaluate the therapeutic potential of drugs and natural products targeting this pathway, and prospect the critical issues needing resolution in future research and clinical translation.

## 2 Molecular mechanisms of Nrf2/HO-1

### 2.1 Suppression of oxidative stress and lipid peroxidation (ferroptosis)

**FIGURE 1:**
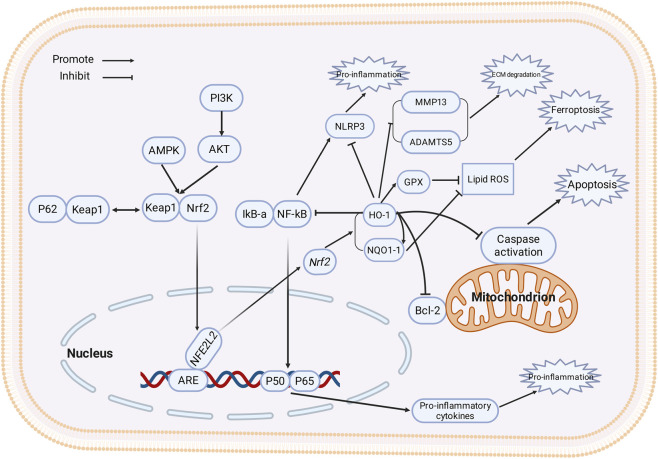
Molecular mechanisms of Nrf2/HO-1 pathway in joint cells of arthritis. Under the action of upstream regulators such as AMPK, PI3K/Akt, and p62, Nrf2 dissociates from Keap1, translocates into the nucleus, and binds to the *ARE*, thereby initiating the transcription of downstream genes including *HMOX1* and *NQO1*. The upregulated Nrf2/HO-1 pathway exerts protective effects through the following mechanisms: (1) Inhibiting lipid peroxidation *via* enzymes such as GPX4 and NQO1, thereby preventing cellular ferroptosis; (2) Antagonizing the transcription and activation of NF-κB and the NLRP3 inflammasome, suppressing the production of pro-inflammatory factors to mitigate inflammation; (3) Downregulating matrix-degrading enzymes MMPs and ADAMTS to preserve the extracellular matrix; (4) Modulating apoptosis by downregulating anti-apoptotic proteins such as Bcl-2 and inhibiting caspase activation.

Oxidative stress is a significant inducer of cellular damage in arthritis pathology. Recent studies have further established ferroptosis—an iron-dependent, lipid peroxidation-driven novel form of cell death—as a key mechanism in chondrocyte degeneration and joint structural destruction. In various OA models, iron overload and increased lipid peroxidation can lead to loss of glutathione peroxidase 4 (GPX4) function, thereby inducing chondrocyte ferroptosis. Activation of the Nrf2/HO-1 pathway enhances the expression of multiple antioxidant enzymes and upregulates intracellular iron homeostasis regulatory proteins, effectively inhibiting lipid ROS accumulation and mitigating ferroptosis-related injury (Zhang Z. et al., 2025; Ruan et al., 2023). For instance, the natural flavonoids baicalein and baicalin significantly reduce interleukin-1β (IL-1β)-induced lipid peroxidation and ferroptosis in chondrocytes *via* the AMP-activated protein kinase (AMPK)/Nrf2/HO-1 axis, thereby delaying OA progression and improving histological scores (Wan et al., 2023). Another study showed that iron chelators, while inhibiting iron-dependent oxidation, synergistically activate Nrf2 signaling, significantly reducing cartilage destruction under inflammatory conditions, highlighting Nrf2’s central role in regulating the “iron metabolism-lipid peroxidation” loop (Tao et al., 2024). These findings not only deepen our understanding of Nrf2’s mechanism in antioxidation and anti-ferroptosis but also provide a theoretical basis for combining iron metabolism intervention with Nrf2 activation in arthritis treatment (Figure 1).

### 2.2 Keap1-Nrf2 release and transcriptional activation mechanism

Under homeostatic conditions, Kelch-like ECH-associated protein 1 (Keap1) acts as a negative regulator of Nrf2, mediating its degradation *via* the ubiquitin-proteasome pathway, maintaining low expression levels. When cells encounter oxidants or electrophilic compounds, key cysteine residues in Keap1 undergo covalent modification, or Keap1 is selectively degraded *via* autophagy, relieving its inhibition on Nrf2. This allows Nrf2 to accumulate in the nucleus, bind to the ARE, and initiate transcription of downstream genes (Zhu et al., 2022; Kedhem et al., 2025). Additionally, accumulation of the autophagy adapter protein p62 can competitively bind Keap1, indirectly promoting Nrf2 stability and activation (Shan et al., 2024); upstream kinases such as AMPK and PI3K/Akt can also influence Nrf2 nuclear translocation and transcriptional activity through phosphorylation (Gong et al., 2025; Liu C. et al., 2024). Recent studies have found that various natural products or synthetic small molecules can effectively activate Nrf2 by downregulating Keap1 expression, promoting p62-mediated Keap1 degradation, or activating upstream kinases, mechanisms validated in protection models of synoviocytes and chondrocytes (Li S. et al., 2024; Fan et al., 2025). Further elucidating the dynamic regulatory mechanisms of the Keap1-Nrf2 interaction will aid in developing more selective Nrf2 activation strategies (Figure 1).

### 2.3 Reciprocal inhibitory network with inflammatory signaling

There exists extensive mutual inhibition between the Nrf2/HO-1 pathway and classical inflammatory signaling pathways, forming a core network regulating joint inflammation. Catalytic products of HO-1, such as CO and biliverdin/bilirubin, can inhibit IKK complex activity or block nuclear translocation of the NF-κB subunit p65, thereby downregulating NF-κB-driven pro-inflammatory gene expression. In OA models, Cucurbitacin B effectively inhibits NF-κB/NLRP3 pathway activation by activating Nrf2/HO-1, significantly slowing cartilage tissue damage (Lou et al., 2024). On the other hand, Nrf2 activation significantly reduces intracellular ROS levels, a key trigger for NLRP3 inflammasome assembly and activation. Studies show that inhibiting Nrf2/HO-1 enhances NLRP3 inflammasome activation and subsequent maturation/secretion of IL-1β/IL-18, exacerbating joint inflammation (Chen et al., 2019). These interactions have been repeatedly verified in various experimental models of gout, OA, and RA, indicating that Nrf2/HO-1 not only directly enhances antioxidant defense but also synergistically reduces tissue damage by suppressing inflammatory signaling (Figure 1).

### 2.4 Regulation of matrix metabolism

Excessive degradation of the extracellular matrix (ECM) is a central link in arthritic cartilage destruction. Nrf2 activation significantly downregulates the expression of various matrix metalloproteinases (MMPs) and ADAMTS family members, thereby inhibiting the breakdown of key matrix components like collagen and aggrecan. Multiple pharmacological studies show that experimental groups with upregulated Nrf2/HO-1 signaling exhibit suppressed MMP-13 and ADAMTS5 expression alongside preserved type II collagen expression. For example, in an OA model, platelet-rich plasma (PRP) downregulated MMP-13 and reduced chondrocyte apoptosis *via* activating the Nrf2/HO-1 pathway (Du et al., 2024). Furthermore, review literature indicates that Nrf2 can indirectly downregulate proteases like MMPs by inhibiting NF-κB activation (Sheng et al., 2024a). These results suggest that the Nrf2/HO-1 system protects cartilage matrix structural integrity through dual “antioxidant” and “anti-inflammatory” mechanisms, providing theoretical support for its application in delaying joint degeneration (Figure 1).

### 2.5 Interplay between programmed cell death pathways (apoptosis and ferroptosis)

Beyond regulating ferroptosis, the Nrf2/HO-1 pathway significantly influences the classical apoptosis program. In the arthritic environment, oxidative stress triggers the initiation of the mitochondrial apoptosis pathway and activates the Caspase cascade, leading to massive chondrocyte loss. Nrf2 activation maintains mitochondrial membrane potential, reduces ROS generation, and upregulates anti-apoptotic molecules like Bcl-2, thereby inhibiting the activation of Caspase-3 and Caspase-9. In an OA model, PRP downregulated the pro-apoptotic factor Bax and Caspase-3 while upregulating Bcl-2 *via* the Nrf2/HO-1 pathway, exerting an anti-apoptotic effect (Du et al., 2024). Moreover, Nrf2’s parallel inhibition of both ferroptosis and apoptosis pathways suggests its key role as an integrator of different cell death signals. For example, Baicalein inhibits chondrocyte ferroptosis *via* the AMPK/Nrf2/HO-1 axis (Liu Y. et al., 2024), whereas interfering with Nrf2/HO-1 exacerbates NOX1(NADPH oxidase 1)-induced ferroptosis and cellular damage (Tao et al., 2024). Future research needs to further dissect Nrf2’s hub role in coordinating multi-modal cell death to expand its application in treating degenerative joint diseases (Figure 1).

### 2.6 Multi-layered integration and plasticity of upstream regulation

The activity of the Nrf2/HO-1 pathway is precisely regulated by multiple upstream signals, including the energy-sensing kinase AMPK, autophagy flux, epigenetic modifications, and non-coding RNAs (e.g., miRNAs and lncRNAs). A recent review by Nan et al. emphasized the high plasticity and dual regulatory nature of this pathway in bone cells (Nan et al., 2025). Simultaneously, different joint cell types (e.g., chondrocytes, synovial fibroblasts, macrophages, osteoclasts) exhibit significant differences in their upstream kinase profiles, iron-handling capacity, and endogenous antioxidant reserves, leading to varied effects from the same Nrf2 activation strategy across different cells (Huang et al., 2025). Yuan et al. also noted that Nrf2/HO-1 is tightly coupled with metabolic/kinase networks, collectively determining cellular stress adaptation capacity (Yuan et al., 2024). These findings suggest that future intervention strategies must fully consider cell type specificity and the dynamic changes across disease stages to achieve precise temporal control and delivery.

In summary, the Nrf2/HO-1 system plays a central protective role in maintaining joint homeostasis through interconnected molecular networks: it directly counters oxidative stress and ferroptosis, while also comprehensively preserving tissue structure and function by inhibiting inflammatory axes like NF-κB/NLRP3 and reducing the inductive expression of matrix-degrading enzymes. However, the pathway’s potential to disrupt iron metabolism balance or affect osteogenic/proliferative processes in certain pathological contexts necessitates careful design of dosage, timing, and delivery strategies in clinical translation.

## 3 Regulatory roles of the Nrf2/HO-1 pathway in cellular and *in vivo* models

### 3.1 Chondrocytes

#### 3.1.1 Key observations and mechanism integration

Chondrocytes reside in a hypoxic, nutrient-poor joint microenvironment and are highly susceptible to producing a burst of ROS under stimulation by inflammatory factors like Interleukin-1β(IL-1β)and Tumor necrosis factor-alpha (TNF-α), subsequently activating cell death and matrix degradation programs. Recent research consistently shows that Nrf2 activation protects chondrocytes and maintains ECM homeostasis through three synergistic pathways (Sheng et al., 2024a): Directly upregulating antioxidant genes like *NQO1*, *GCLC*, and *GPX4* to reduce ROS and lipid peroxidation levels (Lal et al., 2021); Inducing HO-1 expression to generate active molecules like CO and biliverdin/bilirubin, inhibiting pro-inflammatory cascades such as NF-κB/MAPK (Saha, 2024); Regulating iron metabolism-related proteins (e.g., Ferritin heavy chain (FTH1)) to reduce ferroptosis risk. These mechanisms collectively reduce the inductive expression of MMPs/ADAMTS and promote the retention of type II collagen and aggrecan (Lal et al., 2021; Liu J. et al., 2024). For example, baicalein and baicalin significantly inhibit IL-1β-induced chondrocyte ferroptosis and MMP-13 overexpression *via* the AMPK/Nrf2/HO-1 axis (Wan et al., 2023; Liu J. et al., 2024); the small molecule agonist omaveloxolone simultaneously activated *NFE2L2/ARE* and inhibited NF-κB in IL-1β-stimulated chondrocytes, reducing apoptosis and ECM degradation (Jiang et al., 2022a). Latest research also reveals a negative feedback loop between NADPH oxidase family member NOX1 at the ROS source and Nrf2: NOX1 overactivation inhibits Nrf2/HO-1, promoting ferroptosis; inhibiting NOX1 restores Nrf2 function and alleviates tissue damage (Tao et al., 2024). These findings provide solid experimental evidence for targeting Nrf2 signaling to delay cartilage degeneration.

#### 3.1.2 Temporal and dose dependency: from protection to potential risk

Although short-term or moderate Nrf2/HO-1 activation demonstrates clear protective effects, long-term, sustained high HO-1 expression may lead to free Fe^2+^ accumulation. If cells lack sufficient iron sequestration (e.g., insufficient ferritin expression) or iron efflux mechanisms, this free iron can promote lipid peroxidation *via* the Fenton reaction, potentially increasing susceptibility to ferroptosis. This “double-edged sword” effect has been hinted at in some iron overload models and long-term dosing studies, suggesting the need for concurrent monitoring of iron metabolism indicators and consideration of combining iron chelators or ferritin-inducing strategies in clinical translation (Sheng et al., 2024a; Lal et al., 2021). Currently, most chondrocyte research is based on short-term stimulation (IL-1β, H_2_O_2_, erastin) and acute/subacute animal models (e.g., DMM, MIA, ACLT). Understanding of the long-term effects of Nrf2/HO-1 in the context of aging and chronic metabolic comorbidities remains insufficient. Therefore, future research should focus on (Sheng et al., 2024a): Utilizing aging and metabolic syndrome animal models to better simulate the clinical patient population (Lal et al., 2021); Systematically evaluating iron metabolism indicators (serum ferritin, transferrin saturation, tissue iron staining) to comprehensively assess the long-term impact of HO-1 activation on iron homeostasis (Saha, 2024); Comparing the efficacy and safety of short-term high-intensity *versus* long-term low-intensity Nrf2 activation within the same model to determine the optimal therapeutic window. Overall, these findings highlight the importance of precise temporal and dose control of Nrf2/HO-1 activation to maximize chondroprotective effects while avoiding iron-related adverse outcomes.

### 3.2 Fibroblast-like synoviocytes (FLS)

In rheumatoid arthritis, FLS are not only major producers of inflammatory cytokines but also exhibit invasive growth and destructive potential towards cartilage and bone tissue. Nrf2/HO-1 exerts regulatory effects in FLS through two main lines: on one hand, directly inhibiting NF-κB and MAPK signaling, reducing the secretion of IL-6, IL-8, and MMPs; on the other hand, regulating cellular energy metabolism, inhibiting glycolysis-dominated metabolic reprogramming, lowering lactate production and local acidification, thereby disrupting the self-sustenance of the pro-inflammatory microenvironment (Liu et al., 2023; Kaur et al., 2021; Prajapati et al., 2025). Pharmacological studies show that calycosin, 7-deacetyl-gedunin, and some flavonoids can activate Nrf2/HO-1 in RA-derived FLS, simultaneously reducing pro-inflammatory gene expression and cell invasiveness (Chen et al., 2022; Su et al., 2016; Castejón et al., 2017). Additionally, some studies suggest that HO-1-derived CO can regulate FLS migration and metalloproteinase expression *via* the sGC-cGMP signaling axis, indicating that HO-1 metabolites possess rapid, “non-transcriptional” regulatory functions (Liu et al., 2023). Most current FLS studies rely on short-term *in vitro* stimulation (TNF-α, lipopolysaccharide (LPS), H_2_O_2_) or single drug treatments, lacking data from 3D synovial organoids or humanized chronic transplantation models, limiting our understanding of the impact of long-term Nrf2 activation on synovial tissue remodeling (e.g., fibrosis, angiogenesis). Subsequent work is recommended to combine synovial organoids, metabolomics, and single-cell transcriptomics to deeply parse the specific regulatory networks of Nrf2 in different FLS subsets.

### 3.3 Macrophages

Macrophages in arthritis can polarize into pro-inflammatory M1 or anti-inflammatory/reparative M2 phenotypes, whose dynamic balance directly influences disease progression and tissue repair outcomes. Nrf2 activation tends to inhibit M1-associated markers (Inducible nitric oxide synthase (iNOS), IL-1β, TNF-α) and promote the upregulation of M2 signature molecules (Arginase-1 (Arg-1), Cluster of differentiation 206 (CD206)); HO-1 and its product CO directly promote M2 polarization by inhibiting NF-κB and activating pathways like Signal transducer and activator of transcription 6 (STAT6)/Peroxisome proliferator-activated receptor gamma (PPARγ) (Wang and He, 2022). In models like collagen-induced arthritis (CIA), Nrf2 agonists or HO-1 inducers significantly alleviated synovial inflammation and bone erosion while promoting the recruitment and functional activation of reparative macrophages (Zhang L. et al., 2025; Zhendong et al., 2025). On the other hand, ROS is a key trigger for NLRP3 inflammasome activation. Nrf2 reduces Caspase-1 activation and IL-1β/IL-18 secretion by lowering ROS levels and inhibiting Thioredoxin-interacting protein (TXNIP) -NLRP3 interaction, exerting anti-inflammatory effects in crystalline arthritis (e.g., gout) and OA (Yan et al., 2020). It is important to note that intra-articular macrophages can originate from circulating monocytes or tissue-resident cells, and their metabolic characteristics and Nrf2 responses differ: for instance, bone marrow-derived macrophages rely more on glycolysis, while resident macrophages possess greater oxidative metabolic capacity (Viola et al., 2019). This heterogeneity implies that Nrf2 activation may yield different outcomes in different macrophage subsets; excessive suppression of M1 responses might alleviate inflammation but could also impair pathogen clearance or certain repair processes. Therefore, *in vivo* studies should combine cell lineage tracing and single-cell technologies to clarify the specific pathways and spatiotemporal characteristics of Nrf2-driven macrophage phenotype conversion.

### 3.4 Osteoclasts and osteoblasts

The RANKL-RANK-NFATc1 signaling pathway is central to osteoclast differentiation, and oxidative stress can potentiate RANKL downstream signaling, promoting bone resorption. Nrf2/HO-1 activation inhibits osteoclast differentiation and the expression of bone resorption markers (TRAP, Cathepsin K) by suppressing ROS, reducing NF-κB activity, and directly interfering with NFATc1 transcriptional activation (Chao et al., 2025; Ji et al., 2024). In osteoblasts, moderate ROS is necessary for osteogenic differentiation, but excessive ROS inhibits Runx2 activity and matrix mineralization; Nrf2 supports normal osteoblast differentiation and protects them from oxidative damage by maintaining the oxidant-antioxidant balance. However, long-term excessive activation of Nrf2 might also inhibit certain differentiation steps dependent on ROS signaling, indicating the need for precise control of Nrf2 activity intensity when intervening in bone metabolism (Yao et al., 2025). Most current studies are based on *in vitro* induction of differentiation or short-term resorption assays. The net effect of Nrf2 on bone remodeling under long-term loading conditions and its interaction with systemic endocrine and metabolic factors remain poorly understood. Before advancing to the clinic, comprehensive evaluation of Nrf2 activation’s impact on bone density, microstructure, and mechanical properties should be conducted in large animal long-term models, rather than relying solely on molecular markers.

### 3.5 Integrated validation in *in vivo* models: model dependence and translational implications

Commonly used arthritis models include: destabilization of the medial meniscus/anterior cruciate ligament transaction (DMM/ACLT) (trauma/mechanically induced OA), monosodium iodoacetate (MIA) (chemically induced OA), collagen-induced arthritis (CIA) (autoimmune RA), and monosodium urate injection (MSU) (gout model). Overall, pharmacological interventions based on Nrf2 activation demonstrate positive effects in most models, including anti-inflammation, inhibition of ECM degradation, pain relief, and improved structural damage. However, the effect size and duration highly depend on the model type, route of administration (local intra-articular vs. systemic), dose, and timing. For example, in MIA and DMM models, intra-articular injection or sustained-release formulations achieve high local drug retention and tissue protection; whereas systemic administration (oral or intraperitoneal) can modulate systemic immunity/metabolism but carries risks of hepatorenal toxicity and potential oncological concerns (noting Nrf2’s potential role in tumor drug resistance) (Lal et al., 2021; Song et al., 2021; Yan et al., 2020). The joint, as a relatively closed compartment with rapid clearance and poor penetration of synovial fluid, results in short drug retention after local administration. Nanocarriers, exosomes, or hydrogel sustained-release systems can significantly prolong intra-articular drug residence and reduce systemic exposure, representing feasible solutions to overcome pharmacokinetic limitations. Importantly, *in vivo* evidence increasingly suggests that short-term, high-intensity, or pulsed Nrf2 activation during acute inflammatory peaks provides superior protective outcomes compared with long-term continuous stimulation, achieving rapid ROS suppression, acute-phase inflammasome inhibition, and matrix preservation while minimizing risks associated with chronic systemic activation. This insight underscores the translational relevance of designing temporally optimized, flare-responsive dosing strategies rather than uniform long-term regimens (Wu Z. et al., 2023). Major obstacles in translating from models to humans include species differences (differences in immunity and metabolism between mice and humans), insufficient reproduction of “chronic low-grade inflammation” by existing models, and the complex comorbidities (aging, metabolic diseases, polypharmacy) in clinical populations. Therefore, it is recommended to prioritize before entering clinical trials (Sheng et al., 2024a): Validation using humanized synovial/cartilage transplantation or organoid models (Lal et al., 2021); Assessment of intra-articular pharmacokinetic parameters (area under curve, retention time) and long-term toxicity (oncology, immune monitoring) (Saha, 2024); Patient stratification and early efficacy assessment based on biomarkers (*NFE2L2* target gene expression, joint fluid ROS, and ferroptosis markers) (Sun S. et al., 2023; Laragione et al., 2025).

In summary, analysis by cell type reveals that Nrf2/HO-1 forms a multi-layered network within the joint, centered on antioxidation, utilizing HO-1 metabolites and metabolic reprogramming to regulate immune and matrix homeostasis. The protective effect of this pathway depends on cell type, disease stage, and metabolic background. The key to successful translation from bench to bedside lies in developing temporal, localized, and combined regulation strategies: namely, short-term local high-efficiency activation to control acute inflammation, followed by low-intensity maintenance or combination with iron homeostasis/immunomodulatory drugs to avoid long-term side effects.

## 4 Comparative analysis of the role of Nrf2/HO-1 in different types of arthritis

### 4.1 Osteoarthritis

The main pathological drivers of OA include mechanical wear, chronic low-grade inflammation, and metabolic abnormalities. Recent studies consistently confirm that in OA cartilage and synovium, the expression of the Nrf2/HO-1 pathway is activated as an endogenous defense response, protecting matrix structure by inhibiting ROS, attenuating NF-κB- and MAPK-mediated inflammatory transcription, and downregulating MMPs/ADAMTS expression (O'Rourke et al., 2024; Zhang F. et al., 2023). Furthermore, evidence regarding ferroptosis is rapidly accumulating: elevated iron load and decreased GPX4 activity can be detected early in OA. *NFE2L2* participates in inhibiting chondrocyte ferroptosis by regulating *GPX4*, *SLC7A11*, and iron metabolism-related genes, serving as a key defense line for cell survival (Sun S. et al., 2023; Zhang Y. et al., 2025). In the early stages or during sudden mechanical stress phases, short-term Nrf2 activation can rapidly reduce ROS peaks and block the initiation of matrix degradation. However, during long-term degeneration, the Nrf2 pathway may become “exhausted” or exhibit attenuated downstream responses (possibly due to epigenetic silencing or persistent Keap1 upregulation), explaining why advanced OA patients respond poorly to similar antioxidant strategies (Sheng et al., 2024a; Sheng et al., 2024b; Jia et al., 2024). Therefore, Nrf2-targeted therapy for OA is more likely to achieve maximum benefit in scenarios of “early intervention” or “postoperative protection.” Clinical strategies should emphasize local delivery technologies (intra-articular sustained release, nanocarriers) to increase the intra-articular area under the concentration-time curve and reduce systemic exposure; simultaneously, iron metabolism and ferroptosis biomarkers should be monitored to prevent potential iron-related side effects from HO-1 induction (Table 1).

**TABLE 1:** Summary of literature on the regulation of the Nrf2/HO-1 pathway by different compounds/interventions in OA.

Compound/Intervention	Upstream/Downstream targets of Nrf2/HO-1	Key demonstrated effect(s) in model	References
Cervus nippon Temminck, Valencene	Upregulates NQO1	Alleviates cartilage erosion	Sun S. et al. (2025), Chen S. et al. (2023)
Senkyunolide I, Taxifolin, echinacoside, Caffeic acid phenethyl ester Ginkgolide C	Inhibits ROS	Suppresses inflammatory, chondrocyte apoptosis and ECM degradation	Li et al. (2025), Jiang et al. (2023), Tan and Zhang (2022), Sun et al. (2022), Ma et al. (2022)
Hypoxia-preconditioned cartilage progenitor cells		Suppresses chondrocyte and ECM formation	Feng et al. (2023)
PD184352		Anti-inflammatory and antioxidant	Zheng et al. (2023)
Cerium oxide nanoparticles, ROS-responsive nanocarrier		Suppresses chondrocyte apoptosis	Xiong et al. (2023), Jiang et al. (2022b)
Acetyl-11-keto-β-boswellic acid		Alleviates synovial inflammation and fibrotic responses	Zhou et al. (2024)
Dimethyl fumarate		Suppresses inflammatory and chondrocyte apoptosis	Lai et al. (2025)
Bilirubin	Upregulates GPX4	Inhibits chondrocyte ferroptosis	Zhao et al. (2025), Zhao et al. (2024a)
Akebia Saponin D		Anti-inflammatory and antioxidant	Gu et al. (2020)
Forsythoside B; Hederagenin	Inhibits Keap1	Suppresses inflammatory, chondrocyte apoptosis and oxidative stress	Li S. et al. (2024), Shen Y. et al. (2023)
4-methylcatechol		Suppresses inflammatory, chondrocyte apoptosis	Zhendong et al. (2025)
“Tianyu” Pairing		Suppresses inflammatory and oxidative stress	Tang et al. (2025)
Arctiin	AKT phosphorylation	Reduces chondrocyte ferroptosis	Yang et al. (2024)
Betulin		Suppresses inflammatory, chondrocyte apoptosis and ECM degradation	Ren et al. (2021)
SiRNA-NF-κB	Inhibits NF-κB	Suppresses inflammatory, chondrocyte apoptosis; alleviates cartilage erosion	Liao et al. (2024)
Stevioside		Suppresses inflammatory and ECM degradation	Wu J. et al. (2023)
Limonin		Suppresses inflammatory, ECM degradation; alleviates cartilage erosion	Jin et al. (2021)
PUM2	Inhibits NEDD4	Enhances chondrocyte ferroptosis	Meng et al. (2024)
C5AR1, Baicalein	Upregulates AMPK, PI3K, Akt	Inhibits chondrocyte ferroptosis	Wan et al. (2023), Lv et al. (2024)
MSC-Exos	Upregulates GOT1/CCR2	Promote cell growth and osteogenic differentiation of bone cells	Peng et al. (2023)
Melatonin	Inhibits miR-146a	Suppresses inflammatory, chondrocyte apoptosis and ECM degradation	Zhou et al. (2022)

### 4.2 Rheumatoid arthritis (RA)

RA is a systemic disease characterized by persistent autoimmune activation of the synovium, involving autoreactive T/B cells, invasive FLS, and abundant inflammatory mediators. Nrf2/HO-1 plays a dual regulatory role in RA: on one hand, it alleviates synovitis by reducing ROS and inhibiting NF-κB and NLRP3 pathways; on the other hand, it slows bone erosion by promoting macrophage M2 polarization and inhibiting osteoclast activity (Ma et al., 2024; Bilski and Nuszkiewicz, 2025). Multiple animal studies (e.g., CIA model) and pharmacological evidence (using dimethyl fumarate (DMF), HO-1 inducers, and various natural products) demonstrate that Nrf2 activation significantly alleviates joint swelling and tissue destruction (Zhang L. et al., 2025; Guo et al., 2025; Okasha et al., 2025). Unlike OA, the immune-driven nature of RA dictates that Nrf2 activation, while suppressing inflammation, may also affect antigen presentation/immune surveillance. Long-term, strong systemic activation of Nrf2 may, in certain contexts, interfere with immune clearance capabilities or alter drug-metabolizing enzyme expression (Alcaraz and Ferrándiz, 2020), necessitating caution particularly in individuals with a history of cancer or susceptibility to infection. In RA treatment, a more feasible strategy is to use Nrf2 activation as an adjunct immunomodulatory approach, combined with existing biologics (e.g., TNF-α inhibitors, JAK inhibitors) to reduce the dose and side effects of immunosuppressants (Saha, 2024; Lin et al., 2023). Future RA research needs to more finely assess the immunomodulatory consequences of Nrf2 at the level of FLS subsets, synovial microenvironment, and T/B cell responses, particularly utilizing single-cell transcriptomics and humanized immune models to analyze its potential impact on antibody production and immunological memory formation (Bilski and Nuszkiewicz, 2025; Cohen-Kaminsky and Jambou, 2005) (Table 2).

**TABLE 2:** Summary of literature on the regulation of the Nrf2/HO-1 pathway by different compounds/interventions in rheumatoid arthritis.

Compound/Intervention	Upstream/Downstream targets of Nrf2/HO-1	Key demonstrated effect(s) in model	References
Isoliquiritigenin	Inhibits ROS	Suppresses inflammatory, chondrocyte apoptosis and ECM degradation	Hung et al. (2024)
Hyaluronic acid-modified PtPdCo-CQ nanocatalyst		Alleviates synovial hyperplasia, cartilage destruction and inflammatory	Zhang L. et al. (2025)
4-Methylcatechol		Alleviates synovial inflammation and cartilage destruction	Zhendong et al. (2025)
Loganin		Alleviates cartilage destruction	Li M. et al. (2024)
Mesua assamica		Alleviates inflammation, and synovial hyperplasia	Puppala et al. (2024)
Xinfeng capsule		Alleviates inflam mation, oxidative stress, synovial hyperplasia, and cartilage destruction	Sun Y. et al. (2023)
Sinomenine derivatives	Upregulates NQO1	Inhibited osteoclast differentiation and inflammation	Guo et al. (2025)
Asiatic acid	Inhibits NF-κB	Inhibits the proliferation and induces the apoptosis of fibroblast-like synoviocytes	Zhang et al. (2024)
Magnoflorine	Inhibits Keap1	Inhibits apoptosis in fibroblast-like synoviocytes, alleviates inflammation	Shen et al. (2022)

### 4.3 Gouty arthritis (Gout)

Gout attacks are triggered by MSU crystal deposition, leading to NLRP3 inflammasome activation and massive IL-1β release. Recent studies show that Nrf2 activation significantly alleviates MSU-induced acute inflammation and pain behavior by reducing intracellular ROS, stabilizing mitochondrial function, and directly or indirectly inhibiting NLRP3 assembly (Zeng et al., 2024; Lin et al., 2020). In acute MSU injection models, Nrf2 agonists like oltipraz showed rapid anti-inflammatory effects accompanied by downregulation of inflammasome signaling (Tian et al., 2024). Gout often presents with alternating “acute flares” and “chronic tophaceous damage.” Therefore, Nrf2 activation can serve as a rapid anti-inflammatory/antioxidant strategy in the acute phase (especially suitable for local or short-course administration), while in long-term recurrence prevention strategies, attention must be paid to synergy with uric acid synthesis inhibition or excretion-promoting drugs (Herdiana et al., 2025) (Table 3).

**TABLE 3:** Summary of literature on the regulation of the Nrf2/HO-1 pathway by different compounds/interventions in gouty arthritis.

Compound/Intervention	Upstream/Downstream targets of Nrf2/HO-1	Key demonstrated effect(s) in model	References
Limosilactobacillus reuteri, Sirtuin 1	Inhibits ROS	Alleviates inflammation	Qin et al. (2025), Zhao et al. (2024b)
Modified Simiaowan, Monosodium urate crystals	Inhibits NLRP3	Alleviates inflammation	Qin et al. (2024), Jhang et al. (2015)
Hesperidin methylchalcone	Inhibits NF-κB	Reduces oxidative stress, hyperalgesia, and synovitis	Ruiz-Miyazawa et al. (2018)

## 5 Therapeutic strategies targeting Nrf2/HO-1 pathways

In degenerative joint diseases such as osteoarthritis, factors including oxidative stress, chronic inflammation, programmed cell death (apoptosis and ferroptosis), extracellular matrix degradation, and impaired repair capacity of cartilage and bone tissue converge, placing chondrocytes, synovial cells, macrophages, osteoclasts, and osteoblasts in a persistently damaging microenvironment. Therefore, modulating the Nrf2/HO-1 signaling axis, which governs antioxidant defense, anti-ferroptosis, anti-inflammatory, and cytoprotective functions, has become a crucial direction for restoring joint homeostasis, mitigating pathological damage, and promoting cartilage and bone repair.

### 5.1 Natural products

With advancing research, natural products (such as flavonoids, isoflavones, organosulfur compounds, diterpenoids, etc.) have gradually demonstrated potential for joint protection by gently activating Nrf2/HO-1 pathway. These molecules can reduce oxidative stress, inhibit the expression of matrix-degrading factors, and decrease ferroptosis in chondrocytes (Wang et al., 2025). Recent extensive *in vitro* and animal studies indicate that numerous natural compounds activate Nrf2 through multiple mechanisms: first, directly modifying reactive cysteine residues on the Keap1 protein, relieving its inhibition of Nrf2 (Xu et al., 2024; Zhang A. et al., 2023); second, promoting Nrf2 phosphorylation and nuclear translocation by activating upstream kinases (e.g., AMPK, PI3K/Akt, PKC) (Shen et al., 2022; Yue et al., 2024; Lv et al., 2025); third, inducing aggregation of the autophagy adapter protein p62, mediating selective autophagic degradation of Keap1, and indirectly stabilizing Nrf2 (Liao et al., 2021; Zhu et al., 2023; Fan et al., 2018). This “multi-target, weak synergy” characteristic gives natural products broad potential in mitigating inflammatory peaks and restoring redox homeostasis. Natural components represented by Astragalus, resveratrol, and curcumin have been confirmed in OA and RA models to exert antioxidant and anti-apoptotic effects *via* the Nrf2/HO-1 pathway (Jin et al., 2024; Chen et al., 2024; Chen B. et al., 2023). Simultaneously, natural products can also promote the transition from pro-inflammatory M1 phenotype to anti-inflammatory M2 phenotype in synovial cells and synovial macrophages, thereby reducing the release of inflammatory factors and improving the synovitis environment (Wang and He, 2022). The advantages of natural products lie in their low toxicity and multi-pathway synergy, but their limitations include low oral bioavailability and rapid metabolism, leading to insufficient effective concentrations locally in the joint (Pa et al., 2024). Additionally, their complex pharmacokinetics and potential drug interaction risks suggest they are more suitable for use as local delivery formulations or long-term adjuvant therapies (Sezgin Bayindir et al., 2025; Wang K. et al., 2020).

### 5.2 Small-molecule activators

Directly promoting Nrf2 nuclear translocation *via* small-molecule activators can enhance the antioxidant and anti-inflammatory capacity of chondrocytes, thereby significantly alleviating oxidative damage caused by elevated ROS. Activation of Nrf2 by small molecules can markedly inhibit lipid peroxidation induced by inflammatory stimuli, reduce the expression of MMPs and ADAMTS, preserve key ECM components such as collagen II and proteoglycans, and protect chondrocytes from apoptosis and matrix disruption. Representative synthetic small molecules include DMF, CDDO series compounds, and omaveloxolone (Lal et al., 2021; Jiang et al., 2022a; Dong et al., 2022). These molecules typically rapidly activate *NFE2L2* by covalently modifying Keap1 or altering the cellular redox state, thereby upregulating antioxidant genes like *HMOX1*, *NQO1*, and *GCLC* (Guo et al., 2025; Wan et al., 2021; Su et al., 2021). In complete Freund’s adjuvant-induced arthritic rats, DMF significantly improved inflammation scores and cartilage structure, accompanied by upregulation of the Nrf2/HO-1 pathway (Lal et al., 2021); CDDO-Im achieved cartilage protection by enhancing autophagy and inhibiting apoptosis (Dong et al., 2022). However, clinical experience with bardoxolone methyl (CDDO-Me) has raised important safety concerns. In large clinical trials involving patients with chronic kidney disease, bardoxolone methyl was associated with fluid retention, elevations in blood pressure, albuminuria, hypomagnesemia, and unintended weight loss, reflecting a pattern of metabolic and electrolyte imbalance as well as altered vascular and renal regulation (Pergola et al., 2011; Wang et al., 2014; Bondi et al., 2024; de Zeeuw et al., 2013). Therefore, small molecule activation strategies require strict weighing of dose and exposure route. Recently, researchers have proposed improving safety and efficacy through local delivery, prodrug modification, and time-window control (Sezgin Bayindir et al., 2025).

### 5.3 Gene & RNA modulation

Gene and nucleic acid interventions offer new possibilities for precise modulation of the Nrf2/HO-1 pathway. Increasing Nrf2 levels within chondrocytes not only significantly reduces the release of inflammatory factors and ECM degradation and protects cells from apoptosis but also enhances the ability of chondrocytes to resist ferroptosis, making them more tolerant to lipid peroxidation caused by inflammatory stimuli and iron overload. Furthermore, synovial cells and macrophages are also sensitive to nucleic acid modulation; enhancing Nrf2 signaling can suppress inflammatory responses and synovial hyperplasia. For example, using siRNA to knock down Keap1 can relieve Nrf2 inhibition in synoviocytes, reducing ROS generation and inflammatory factor release. Furthermore, non-coding RNAs such as siRNA and circRNA targeting Nrf2 or Keap1 have also shown protective effects in arthritis models (Yang et al., 2018; Fu et al., 2025; Luo et al., 2022; Sun L. et al., 2025). Gene editing tools (e.g., CRISPRa) can directly enhance the promoter activity of *NFE2L2* or *HMOX1*, showing promise to further expand the therapeutic window (Lee et al., 2017; Kim et al., 2021). A series of related studies have demonstrated that miRNAs are abnormally expressed in chondrocytes and synovial fibroblasts in arthritis, and they indirectly or directly regulate the activity and expression of Nrf2 through interaction with the NF-κB pathway, thereby affecting cellular oxidative stress balance, inflammatory responses, and apoptosis processes (Cheleschi et al., 2023; Cheleschi et al., 2019a; Cheleschi et al., 2019b). Specifically, miR-34a has been confirmed to directly target the 3′-UTR region of *NFE2L2* mRNA, suppressing *NFE2L2* expression and the transcription of its downstream antioxidant genes such as *SOD-2* and *CAT*, thereby exacerbating oxidative stress and apoptosis. MiR-146a, on the other hand, indirectly influences Nrf2 activation by regulating downstream molecules of the NF-κB pathway such as IRAK1 and TRAF6; under stimulation by IL-1β or visfatin, upregulation of miR-146a expression can inhibit the Nrf2-mediated antioxidant response. While miR-181a plays a role in regulating apoptosis and oxidative stress, its direct effect on Nrf2 remains unclear; it may indirectly affect cellular antioxidant capacity by modulating mitochondrial metabolism and BCL2 family proteins. miR-210 is elevated in the synovial fluid of OA patients and influences inflammation and oxidative stress responses by modulating NF-κB signaling, potentially indirectly interfering with the protective function of the Nrf2 pathway. Notably, the expression of these miRNAs is regulated by adipokines such as visfatin and resistin as well as inflammatory factors such as IL-1β, and their effects can be reversed by NF-κB inhibitors or miRNA-specific inhibitors. Therefore, targeting these miRNAs or their upstream regulatory pathways may represent a novel strategy for enhancing Nrf2-mediated cytoprotective effects and alleviating the pathological progression of arthritis. However, the dense ECM of joint tissues poses a severe challenge for nucleic acid drug delivery. Non-viral nanocarriers or exosome-encapsulated nucleic acid systems show good local retention and biocompatibility (Sezgin Bayindir et al., 2025; Luo et al., 2024). Nonetheless, the long-term safety, immunogenicity, and reversibility of expression regulation of nucleic acid therapies still require in-depth validation.

### 5.4 Nano- and exosome-based delivery

Regarding drug delivery systems, the joint cavity, as a locally enclosed microenvironment, makes nanoparticles and exosomes ideal platforms for delivering Nrf2 activators or nucleic acid-based drugs. Surface modification or the construction of release systems responsive to inflammatory or oxidative stimuli can significantly increase drug retention time in the joint cavity, allowing fuller action on chondrocytes, synovial cells, macrophages, and further extending to osteoclasts and osteoblasts at the bone-cartilage interface. This approach can not only synchronously regulate oxidative stress, inflammation, and cell death processes but also coordinate ECM metabolism and bone remodeling. Furthermore, exosomes derived from mesenchymal stem cells themselves carry miRNAs and proteins with anti-inflammatory and tissue-repairing properties, creating a dual effect with the loaded Nrf2-modulating molecules, thereby more effectively promoting cartilage repair and bone reconstruction. Polymer nanoparticles, liposomes, and metal-organic frameworks can achieve targeted localization to cartilage or synovium through surface modification (He et al., 2025). Encapsulating quercetin or DMF using such systems significantly prolonged drug retention and enhanced anti-inflammatory effects in experimental OA models (Selvadoss et al., 2024). Exosomes, as natural nanocarriers, also perform prominently in regulating Nrf2 signaling: mesenchymal stem cell-derived exosomes can transmit HO-1 upregulation signals and promote cartilage repair (Ma et al., 2024). However, this strategy still faces challenges such as production standardization, loading efficiency, dose control, and immunocompatibility, especially the safety of long-term repeated administration requires systematic evaluation (Lu et al., 2023; Singer et al., 2024; Pînzariu et al., 2025). Future efforts should focus on developing composite platforms combining exosomes and biodegradable nanomaterials to achieve unified sustained release, targeting, and biodegradability.

### 5.5 Combinatorial & precision approaches

Against the backdrop of multiple pathological factors collectively driving joint degeneration, a single Nrf2 activation strategy is often insufficient to comprehensively reverse the disease course. Therefore, combination therapy is gradually becoming a necessary development direction. By combining Nrf2 activation with anti-ferroptosis compounds, anti-inflammatory drugs, matrix-protective agents, or bone remodeling modulators, synergistic effects can be simultaneously produced on multiple key cell types. Protecting chondrocytes from oxidative and ferroptosis damage while alleviating the pro-inflammatory phenotype of synovial macrophages and regulating osteoclast and osteoblast processes to improve the osteochondral interface may achieve comprehensive improvement of the entire joint ecosystem. For example, Combining Nrf2 activators with ferroptosis inhibitors (e.g., ferrostatin-1) or anti-inflammatory drugs (e.g., Janus kinase (JAK) inhibitors) can control cytokine storms while preventing ferroptosis (Ni et al., 2023). Furthermore, combination with metabolic modulators (e.g., metformin or SIRT1 activators) can enhance the antioxidant response and improve energy homeostasis through AMPK-Nrf2 cross-talk (McCarty et al., 2022). In terms of precision medicine, it is recommended to stratify patients based on joint fluid HO-1 levels (Kitamura et al., 2011; Andersen et al., 2025), lipid peroxidation markers (Wang C. C. et al., 2020; Biniecka et al., 2010), and transcriptomic features (Lee et al., 2023; Mao et al., 2024) to determine the optimal activation intensity and dosing timing.

**FIGURE 2:**
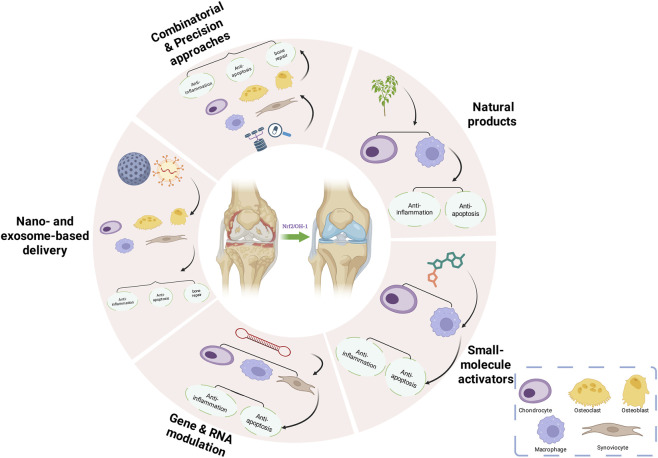
Therapeutic strategies targeting Nrf2/HO-1 pathways.

Based on existing evidence, we propose a three-step strategy for clinical translation: First, achieve local high-efficiency, short-term Nrf2 activation using nano or exosome delivery platforms to minimize systemic exposure risk; Second, adopt combination regimens pairing Nrf2 activation with ferroptosis inhibition or anti-inflammatory drugs to dynamically regulate the redox-immune balance; Third, establish a biomarker system centered on HO-1, NQO1 expression, and oxidative stress markers to enable patient stratification and individualized treatment. Overall, the Nrf2/HO-1 pathway possesses both mechanistic rationale and pharmacological plasticity in arthritis intervention. Its future successful translation depends on the maturation of delivery platforms, optimization of dose-timing relationships, and systematic safety evaluation (Figure 2).

## 6 Discussion and future perspectives

The Nrf2/HO-1 axis, as a potential target for arthritis treatment, has demonstrated clear biological rationale and therapeutic promise in numerous basic and translational studies (Ma et al., 2024). However, multiple critical obstacles remain on the path from “proof-of-concept” to “clinical practice.” To promote the translation into high-reliability, low-risk clinical therapies, this section discusses key issues with corresponding technical/trial design countermeasures, proposing an actionable research and development roadmap.

### 6.1 Temporal and dose window uncertainty

The biological effects of Nrf2/HO-1 exhibit significant temporal dependence: short-term activation is often protective, whereas long-term systemic activation may lead to iron metabolism disorders, metabolic reprogramming, and even affect drug-metabolizing enzymes and proliferation signals, posing potential metabolic or oncological risks (Huang et al., 2025). This complex time-effect relationship necessitates precise definition of the treatment “when” and “how much,” for instance, by systematically comparing the differences in long-term outcomes between short-term trigger-based and long-term maintenance dosing regimens (Duffull et al., 2025). To establish a scientifically reliable dosing regimen, we recommend designing animal PK/PD matrix experiments in the preclinical phase that include both short-term pulse and long-term maintenance dosing modes, thereby generating precise time-dose-effect curves to provide a conservative and reliable dose window basis for early clinical trials (Carrara et al., 2025). This systematic research approach will help determine the optimal therapeutic window, maximizing the protective effects of the Nrf2/HO-1 pathway while minimizing potential risks.

### 6.2 Technical bottlenecks in tissue and cell-specific delivery

The joint, as a complex and relatively enclosed physiological microenvironment, with its dynamic circulation of synovial fluid, the barrier effect of dense cartilage matrix, and the structural characteristics of synovial tissue, collectively limit the effective concentration and retention time of drugs at the lesion site after systemic administration. Although nanocarriers, exosomes, and smart responsive hydrogels have shown potential in animal models to prolong drug residence and improve tissue penetration, their clinical translation still faces a series of technical challenges. To overcome these delivery bottlenecks, we should focus on developing more advanced local delivery technologies. This requires prioritizing the development of local delivery platforms responsive to the inflammatory microenvironment or specific temporal signals, such as injectable responsive hydrogels or targeted nano-exosome composite systems, and systematically evaluating their batch consistency during scale-up production, biodegradability, and long-term immunocompatibility in large animal models (Ren et al., 2024). These technological breakthroughs will significantly improve drug targeting and retention time in joint tissues, creating favorable conditions for effective activation of the Nrf2/HO-1 pathway.

### 6.3 Reverse effect of iron metabolism and ferroptosis risk

The catalytic products of HO-1 include free Fe^2+^. If HO-1 upregulation occurs without sufficient simultaneous iron sequestration (e.g., ferritin) or effective iron efflux mechanisms, these free iron ions may exacerbate lipid peroxidation *via* the Fenton reaction, thereby inducing ferroptosis. This reverse effect may completely offset the protective effects brought by Nrf2 activation and even aggravate tissue damage. Several experimental studies have emphasized the importance of ferroptosis in joint degeneration and warned of this double-edged sword effect (Han et al., 2024). To guard against this potential risk, we need to incorporate iron metabolism and lipid peroxidation markers (such as blood/joint fluid ferritin, transferrin saturation, MDA, 4-HNE, GPX4 expression, etc.) as mandatory indicators in all preclinical long-term dosing trials and early clinical follow-ups; simultaneously, actively validate the comprehensive impact of combination regimens of Nrf2 activators and ferroptosis inhibitors on efficacy and safety in animal models (Singer et al., 2024). Through this comprehensive safety assessment strategy, potential iron metabolism disturbances can be identified and intervened early, ensuring treatment safety and effectiveness.

### 6.4 Model differences and insufficient predictive value

Most current evidence comes from acute or subacute disease models in mice or rats (e.g., DMM, MIA, CIA, MSU injection). These models have inherent differences from humans in immune response, systemic metabolic status, and joint anatomy, making them particularly difficult to simulate human senile chronic OA or complex disease progression in the context of metabolic comorbidities, which greatly limits the predictive value of preclinical data for clinical trial outcomes. To enhance the predictive value of preclinical research, we should increase the weight of aging models, metabolic syndrome comorbidity models, and large animal long-term follow-up models in the research system, and integrate the PK/PD parameters, dynamic biomarker profiles, and histological data obtained from these more clinically relevant models as important reference thresholds for deciding whether a candidate drug enters clinical trials. This multi-level, multi-species validation strategy will significantly improve the success rate of preclinical data translation to the clinic and reduce the risk of clinical trial failure.

### 6.5 Lack of biomarker and patient stratification systems

Achieving precise application of Nrf2/HO-1 targeted therapy urgently requires the establishment of biomarker panels capable of reflecting pathway activity and body redox/iron status in real-time. However, standardized biomarker combinations (e.g., joint fluid HO-1, NQO1 mRNA/protein, MDA, 4-HNE, serum ferritin and transferrin saturation) that can be used for patient stratification and early efficacy judgment are still in the exploratory stage. To fill this gap, we recommend proactively exploring and validating biomarkers in early clinical trials. Ideally, an early phase I/IIa trial design should set a carefully selected “minimum viable biomarker panel” as a primary or key secondary endpoint, and embed dynamic sampling at multiple preset time points to capture early response signals after drug treatment, thereby providing evidence-based basis for patient stratification and individualized dosing in subsequent phase IIb/III large-scale trials. This prospective biomarker research strategy not only aids in achieving precision medicine but also provides key decision-making basis for drug development.

### 6.6 Broad spectrum requirements for safety monitoring

Given that the *NFE2L2* transcription factor is involved in regulating numerous metabolic and drug-metabolizing enzyme genes, its long-term, systemic activation may produce widespread biological effects. Therefore, early human trials targeting this pathway must expand the scope of safety monitoring, adopting a broader perspective than conventional trials. Beyond standard imaging and joint function assessments, the research protocol should include longitudinal monitoring of cardiometabolic parameters, liver and kidney function, tumor-related markers, and systemic immune parameters. Particular vigilance is required for potential immune reactions triggered by nucleic acids or exosome vectors (the historical safety event of bardoxolone serves as an important warning). To address these potential risks, prospective trial design and proactive regulatory communication are particularly important. We should preset long-term follow-up plans in the clinical trial protocol (recommended for at least 1–3 years, focusing on cardiovascular and oncological events), and communicate early with regulatory agencies regarding quality control standards and immunological assessment requirements for novel delivery platforms to fully meet regulatory expectations for the safety evaluation of innovative therapies.

## 7 Conclusion

The Nrf2/HO-1 pathway, as a key hub regulating the oxidative stress-inflammation axis, demonstrates significant protective effects in various arthritis pathological processes, including OA and RA. Therapeutic strategies based on this pathway (including natural products, small-molecule activators, nucleic acid regulation, and nano-delivery) show promising tissue protection and anti-inflammatory potential in preclinical research. However, their cell-specific effects, long-term safety, pharmacokinetic properties, and clinical translation pathways still require in-depth exploration. Future research should focus on: elucidating the temporal dependency mechanisms of Nrf2 in different cell types and disease stages; developing more precise and safe targeted delivery systems; establishing predictive biomarker systems; and conducting rigorously designed early clinical trials to verify their efficacy and safety. Comprehensive application of multi-scale (from molecular to clinical) and interdisciplinary research strategies will vigorously promote the Nrf2/HO-1 pathway to become a viable target for arthritis intervention.

## Glossary

4-HNE: 4-Hydroxynonenal
ACLT: Anterior cruciate ligament transection
ADAMTS: A disintegrin and metalloproteinase with thrombospondin motifs
AMPK: AMP-activated protein kinase
ARE: Antioxidant response element
Arg-1: Arginase-1
Bax: Bcl-2-associated X protein
Bcl-2: B-cell lymphoma 2
Caspase: Cysteine-aspartic acid protease
CAT: Catalase
CD206: Cluster of differentiation 206
CIA: Collagen-induced arthritis
circRNA: Circular RNA
CO: Carbon monoxide
CRISPRa: CRISPR activation
DMM: Destabilization of the medial meniscus
DMF: Dimethyl fumarate
ECM: Extracellular matrix
FLS: Fibroblast-like synoviocytes
FTH1: Ferritin heavy chain 1
GCLC: Glutamate-cysteine ligase catalytic subunit
GPX: Glutathione peroxidase
GPX4: Glutathione peroxidase 4
HIF-1α: Hypoxia-inducible factor 1-alpha
HO-1: Heme oxygenase-1
IL-1β: Interleukin-1β
iNOS: Inducible nitric oxide synthase
JAK: Janus kinase
Keap1: Kelch-like ECH-associated protein 1
lncRNA: Long non-coding RNA
LPS: Lipopolysaccharide
MAPK: Mitogen-activated protein kinase
MDA: Malondialdehyde
MIA: Monosodium iodoacetate
miRNA: MicroRNA
MMPs: Matrix metalloproteinases
MSU: Monosodium urate
NFATc1: Nuclear factor of activated T-cells 1
NF-κB: Nuclear factor-kappa B
NLRP3: NLR family pyrin domain containing 3
NOX1: NADPH oxidase 1
NQO1: NAD(P)H quinone oxidoreductase 1
Nrf2: Nuclear factor erythroid 2-related factor 2
OA: Osteoarthritis
PI3K/Akt: Phosphatidylinositol 3-kinase/protein kinase B
PK/PD: Pharmacokinetic/pharmacodynamic
PPARγ: Peroxisome proliferator-activated receptor gamma
PRP: Platelet-rich plasma
RA: Rheumatoid arthritis
RANKL: Receptor activator of nuclear factor kappa-B ligand
RORγt: RAR-related orphan receptor gamma
ROS: Reactive oxygen species
Runx2: Runt-related transcription factor 2
SIRT1: Sirtuin 1
siRNA: Small interfering RNA
SOD: Superoxide dismutase
STAT6: Signal transducer and activator of transcription 6
TNF-α: Tumor necrosis factor-alpha
TRAP: Tartrate-resistant acid phosphatase
TXNIP: Thioredoxin-interacting protein
